# Phenotypic and genomic analysis of the hypervirulent methicillin-resistant *Staphylococcus aureus* ST630 clone in China

**DOI:** 10.1128/msystems.00664-24

**Published:** 2024-08-19

**Authors:** Junhong Shi, Yanghua Xiao, Li Shen, Cailing Wan, Bingjie Wang, Peiyao Zhou, Jiao Zhang, Weihua Han, Fangyou Yu

**Affiliations:** 1 Department of Clinical Laboratory, Shanghai Pulmonary Hospital, School of Medicine, Tongji University, Shanghai, China; 2 Jiangxi Provincial Key Laboratory of Respiratory Diseases, Jiangxi Institute of Respiratory Diseases, The Department of Respiratory and Critical Care Medicine, The First Affiliated Hospital, Jiangxi Medical College, Nanchang University, Nanchang, China; Zhejiang University School of Medicine, Hangzhou, Zhejiang, China

**Keywords:** methicillin-resistant *Staphylococcus aureus*, ST630, genomic evolution, epidemic, virulence

## Abstract

Methicillin-resistant *Staphylococcus aureus* (MRSA) sequence type 630 (ST630) is a rarely reported lineage worldwide. This study aimed to trace the dissemination of the emerging MRSA ST630 clones in China and investigate their virulence potential. We collected 22 ST630-MRSA isolates from across China and performed whole-genome sequencing analysis and virulence characterization on these isolates. Epidemiological results showed that MRSA ST630 isolates were primarily isolated from pus/wound secretions, mainly originating from Jiangxi province, and carried diverse virulence and drug resistance genes. Staphylococcal cassette chromosome mec type V (SCCmec V) predominated (11/22, 50.0%) among the MRSA ST630 isolates. Interestingly, nearly half (45.5%) of the 22 ST630-MRSA isolates tested lacked intact SCCmec elements. Phylogenetic analysis demonstrated that ST630-MRSA could be divided into two distinct clades, with widespread dissemination mainly in Chinese regions. Five representative isolates were selected for phenotypic assays, including hemolysin activity, real-time fluorescence quantitative PCR, western blot analysis, hydrogen peroxide killing assay, blood killing assay, cell adhesion and invasion assay, and mouse skin abscess model. The results showed that, compared to the USA300-LAC strain, ST630 isolates exhibited particularly strong invasiveness and virulence in the aforementioned phenotypic assays. This study described the emergence of a highly virulent ST630-MRSA lineage and improved our insight into the molecular epidemiology of ST630 clones in China.

**IMPORTANCE:**

Methicillin-resistant *Staphylococcus aureus* (MRSA) sequence type 630 (ST630) is an emerging clone with an increasing isolation rate in China. This study raises awareness of the hypervirulent MRSA ST630 clones in China and alerts people to their widespread dissemination. ST630-staphylococcal cassette chromosome mec V is a noteworthy clone in China, and we present the first comprehensive genetic and phenotypic analysis of this lineage. Our findings provide valuable insights for the prevention and control of infections caused by this emerging MRSA clone.

## INTRODUCTION

Methicillin-resistant *Staphylococcus aureus* (MRSA) is a notorious pathogen associated with high incidence rates and mortality (1–3). The significant threat of *S. aureus* infection to human beings is mainly due to the rapid emergence of antibiotic resistance and highly virulent isolates (4–6). In the past few decades, MRSA has spread worldwide as one of the most important pathogens, causing a wide range of infections, from mild skin and soft tissue infections to severe invasive diseases such as sepsis, endocarditis, and necrotizing pneumonia (1–3).

The global epidemiological landscape of MRSA is constantly evolving, with the emergence of new epidemic clones contributing to the burden of infections. While certain MRSA lineages, such as sequence type 5 (ST5), ST59, and ST239, have been dominant in the past, the frequency of these clones has undergone significant changes in recent years (7, 8). Notably, ST630 has became one of the most popular MRSA lineages in Denmark in 2018 (9) and has been identified as an emerging clone in both Denmark and China (10). Li et al. reported that in a tertiary hospital, the proportion of ST630-MRSA among ST630 *S. aureus* clones in China significantly mounted from 0% (2008) to 57% (2017) (8). However, the overall prevalence of ST630-MRSA in China remains low, highlighting the need for a comprehensive investigation of this emerging lineage.

Recent studies have revealed that the staphylococcal cassette chromosome mec (SCCmec) elements of the ST630 strain are extremely similar to that of coagulase-negative staphylococci (CoNS), suggesting an evolutionary connection between them, indicating ST630 strain could obtain glycerol phosphate wall teichoic acid (GroP-WTA) biosynthesis genes from CoNS (9). Furthermore, the occurrence of highly conserved SCCmec elements containing CRISPR-Cas (clustered regularly interspaced short palindromic repeats and CRISPR-associated proteins) in ST630 strains could be related to their unusual composition of cell wall teichoic acids, which has been proposed to enable horizontal gene transfer between CoNS and *S. aureus* (11). However, these studies have not yet conducted an in-depth analysis of the virulence and genomic characteristics of ST630-MRSA.

In the present study, we sequenced 22 ST630-MRSA isolates from five provinces in China by utilizing the Illumina NovaSeq platform. The virulence potential of ST630 isolates was analyzed *in vitro* and *in vivo*. The dissemination of ST630 clone was explored by phylogenetic analysis. We aimed to delineate the genetic features and virulence characteristics of the ST630 clone, providing valuable insights for the development of infection prevention and control measures.

## MATERIALS AND METHODS

### Bacterial isolates and growth conditions

We collected and analyzed a total of 643 non-duplicated clinical MRSA isolates from seven provinces/municipalities in China between 2014 and 2021, from which the epidemiological data of 565 clinical MRSA isolates (including 22 ST630 isolates) between 2014 and 2020 have been published in our previous study (12). Among 643 MRSA isolates, 22 ST630-MRSA isolates were identified by whole-genome sequencing (WGS). These 22 ST630-MRSA strains were confirmed with cefoxitin disk diffusion test. The Illumina sequences of the 22 ST630 isolates are available in NCBI (accession number: PRJNA1125655). CA-MRSA clone USA300-LAC was used as the positive hypervirulent control strain. In this study, *S. aureus* isolates were cultivated in tryptic soy broth (TSB) (Oxoid) and incubated with shaking at 220 rpm in a 37°C incubator.

### Whole-genome sequencing and analysis

WGS was conducted on 22 strains of ST630-MRSA utilizing the Illumina NovaSeq platform. The raw reads were quality-filtered using fastp version 0.20.1 (13). *De novo* assembly of the WGS data was performed with Unicycler version 0.5.0 (14). The assembled genomes of *S. aureus* were subjected to multilocus sequence typing (MLST) using MLST v.2.19.0 (https://github.com/tseemann/mlst). The SCCmec types were ascertained utilizing SCCmecFinder (15). The presence of virulence and resistance genes was determined through ABRicate v1.0.0 (https://github.com/tseemann/abricate), drawing on the virulence factor database (16) and the Comprehensive Antibiotic Resistance Database (17). To elucidate the phylogenetic relationships among the ST630 strains in this study and those from other investigations, genomic sequences of *S. aureus* were retrieved from NCBI RefSeq (data cutoff date: October 2023), resulting in 31 ST630 genomic sequences. Core genome single nucleotide polymorphism (SNP) analysis of 53 ST630 isolates was conducted using Snippy version 4.6.0 and Gubbins version 2.3.4, employing *S. aureus* MR254 (GenBank accession number: GCF_020150915) as the reference (18). Data visualization and annotation were executed with the Interactive Tree Of Life (19).

### Antimicrobial susceptibility testing

The antimicrobial susceptibility of a total of 13 antimicrobial agents was evaluated. For ceftaroline, erythromycin, tetracycline, and ciprofloxacin, susceptibility testing was performed using the disk diffusion method on Mueller-Hinton agar plates (Oxoid, UK). The microdilution broth method was used to evaluate the MICs of gentamicin, daptomycin, mupirocin, rifampin, teicoplanin, linezolid, fusidic acid, vancomycin, and dalbavancin. All antimicrobial susceptibility testing and interpretative criteria were according to break-points mentioned in the Clinical Laboratory Standards Institute guidelines (2020). *S. aureus* ATCC 25923 and ATCC 29213 served as controls for the disk diffusion method and MIC testing, respectively.

### Hemolysin activity assay

The ST630 isolates and the USA300 strains were grown in TSB medium for 24 h and supernatants were collected by centrifugation at 5,000 rpm for 2 min with optical density values normalized at 600 nm. Bacterial supernatant (100 µL) was mixed with 900 µL of phosphate-buffered saline (PBS) containing 3% sterile defibrillation rabbit blood cells and then incubated the mixtures at 37°C for 1 h to assess the hemolytic activity. Triton X-100 served as the positive control, and 0.9% (wt/vol) NaCl was set as the negative control. After centrifugation, the supernatant was collected and measured at 600 nm. Experiments were repeated four times.

### Real-time fluorescence quantitative PCR

The ST630 isolates and the USA300 strains were cultured for 24 h in TSB with shaking (220 rpm) at 37°C. Total RNA was isolated using a Total RNA Purification Kit (Sangon Biotech, Shanghai, China) following the manufacturer’s protocol. Total RNA was reverse transcribed into cDNA using the PrimeScript RT reagent kit with gDNA Eraser (Takara Bio, Inc.). The USA300-LAC strain was used as a control, and *gyrB* was used as the internal reference gene. Real-time quantitative PCR (RT-qPCR) was performed using TB Green Premix Ex Taq II (Takara) on QuantStudio 5 Real-Time PCR System (Applied Biosystems). RNA expression levels of *hla* were calculated by the formula 2^−ΔΔCt^. The primer pairs are shown in Table 1. Experiments were performed thrice.

**TABLE 1:** Primer pairs used in RT-PCRs

Primer	Primer sequence (5′ → 3′)
hla-F	AGCGAAGTCTGGTGAAA
hla-R	AACTAGAAATGGCTCTATGA
gyrb-F	ACATTACAGCAGCGTATTAG
gyrb-R	CTCATAGTGATAGGAGTCTTCT

### Western blot analysis

Western blots were performed as previously described (20). Briefly, five randomly selected ST630 isolates and the USA300 strains were grown in TSB with shaking for 24 h, and bacterial culture supernatants were collected. The total amount of proteins was maintained consistently in loading for SDS-PAGE among the five ST630 and USA300 strains. The protein samples were mixed with Omni-Easy Protein Sample Loading Buffer (EpiZyme Biotechnology Co., Ltd., Shanghai, China) and then denatured at 95°C for 10 minutes. The samples were separated by 12.5% SDS-PAGE and transferred onto a polyvinylidene fluoride (PVDF) membrane. After blocking with 5% bovine serum albumin at room temperature for 3 h, the membrane was incubated at 4°C overnight with a rabbit-derived primary anti-Hla IgG antibody (Sigma) at a dilution of 1/2,500. Subsequently, the membrane was incubated with a goat-derived secondary anti-rabbit antibody (Biosharp) at room temperature for 2 h at a dilution of 1/5,000. The Omni ECL Pico Photoluminescence Kit (EpiZyme Biotechnology Co., Ltd., Shanghai, China) is used for developing images.

### Hydrogen peroxide killing assay

The ST630 isolates and the USA300-LAC strains were grown in TSB for 24 h with shaking (220 rpm) at 37°C. The overnight cultures were washed twice with PBS and then diluted to a concentration of 1 × 10^6^ CFU/mL. A 500 µL aliquot of bacterial suspension was mixed with hydrogen peroxide (H_2_O_2_) to a final concentration of 1 mM. The mixtures were then incubated at 37°C for 1 h with shaking at 220 rpm. The reaction was terminated by the addition of 1,000 U/mL of exogenous catalase (Sigma-Aldrich). After that, the cells were serially diluted with PBS and spread on the trypticase soy agar (TSA) plates. After incubation at 37°C for 24 h, the viable cells were calculated to assess the sensitivity of ST630 isolates to H_2_O_2_. All tests were repeated four times.

### Human whole-blood killing assay

The ST630 isolates and the USA300-LAC strains were grown in TSB for 24 h with shaking (220 rpm) at 37°C. Overnight cultures were centrifuged at 12,000 rpm for 1 min at room temperature and adjusted to a concentration of 1 × 10^6^ CFU/mL using sterile PBS. The bacterial suspension was gently mixed with the whole blood of healthy volunteers in a 1:1 ratio. Afterward, the mixtures were incubated at 37°C for 1 h with shaking (220 rpm) and bacterial viability was determined by plating dilutions on TSA plates. All experiments were repeated four times.

### Cell adhesion assay

The A549 lung epithelial cells and RAW264.7 murine macrophage-like cells are derived from the preserved cells in our laboratory. They were grown in Dulbecco's modified Eagle medium (DMEM) after supplementing with 10% fetal bovine serum at 37°C and 5% CO_2_. About 5 × 10^5^ cells were seeded into 12-well plates. Before use, the plates were washed twice with PBS. Bacterial cultures were incubated for 16 h at 37°C to the logarithmic growth phase and resuspended in DMEM without serum. Furthermore, the cells were infected with bacteria (multiplicity of infection [MOI] = 10:1) and co-incubated at 37°C for 2 h. Subsequently, the supernatant was aspirated and discarded, and the plates were washed three times with sterile PBS to remove loosely adherent bacteria. Then, cells were dissociated with 200 µL trypsin-EDTA for 3 min at 37°C. A549 cells were lysed with 0.05% Triton X-100. Bacterial CFU was determined by plating serial dilutions of epithelial cell lysates onto TSA plates.

### Cell invasion assay

About 5 × 10^5^ cells A549 and RAW264.7 cells were seeded into 12-well plates, respectively. Before use, the plates were washed twice with PBS. Bacterial cultures were incubated for 16 h at 37°C to the logarithmic growth phase and resuspended in DMEM medium without serum. Secondly, the cells were infected with bacteria (MOI = 10:1) and co-incubated at 37°C for 1 h. Following incubation for 1 h, infected cells were washed three times with PBS before the addition of 10% (vol/vol) DMEM supplemented with 10 µg/mL lysostaphin (Sigma-Aldrich) and 100 µg/mL gentamicin (Sigma-Aldrich) to each well. Later, plates were incubated for 1 h to kill extracellular bacteria. Following incubation, the cells were washed with PBS and further incubated in fresh 10% (vol/vol) FCS-DMEM. At 12 h post-infection, infected cells were washed three times with PBS to remove extracellular bacteria and dead cells and lysed by the addition of 0.5% (vol/vol) Triton X-100 (Sigma-Aldrich). The number of intracellular bacteria CFU was determined by serial dilution and plating on TSA agar.

### Mouse skin abscess model

BALB/C mice (6 weeks old, female) were randomly divided into six groups (*n* = 5 per group). ST630 isolates and the USA300-LAC were grown overnight in TSB. The overnight cultures in a 200-fold dilution were inoculated into TSB for 9 h (the post-exponential phase) and washed three times with PBS. Then, 100 µL PBS containing 1 × 10^7^ suspended bacterial cells was injected subcutaneously into the shaved flanks of mice. The sizes of lesions and abscesses were monitored daily with calipers, and the calculation formula was as follows: *a* = π × (*L* × *W*). Three days after the skin abscesses were measured and recorded, the mice were euthanized and their skins were dissected. The bacterial load of abscess homogenates was determined by serial dilution and culture on TSA plates.

### Statistical analysis

All analyses were performed using GraphPad Prism 8 (GraphPad Software Inc., San Diego, CA, USA). Results derived from samples between two groups were treated with unpaired two-tailed Student’s *t*-test and χ^2^ test. *P* < 0.05 was statistically considered to be significant. Error bars in the figures represented the standard deviation of a data set (mean ± standard). *P < 0.05, **P < 0.01, ***P < 0.001, and ****P < 0.0001.

## RESULTS

### Identification of MRSA ST630

In this study, we collected a total of 565 MRSA isolates retrospectively collected at seven representative hospitals between 2014 and 2020. The MRSA isolates covered seven geographical districts scattered across mainland China. Sequence types of MRSA strains were confirmed by MLST. Overall, 22 MRSA clinical isolates verified to be ST630 were identified from the seven collections, accounting for 3.8% (22/565). The majority of ST630-MRSA isolates were detected in Jiangxi, representing 40.9% (9/22), while other isolates were from different regions, including Sichuan (4/22), Shanghai (4/22), Zhejiang (3/22), and Hubei (2/22). Most of the ST630 isolates were identified as elderly people and young children, together accounting for 77.3% (17/22). Our isolates comprised mixed specimen types. ST630 accounted for 5.1% (10/195) of pus/wound exudate samples, 3.9% (7/178) of blood samples, and 2.6% (5/192) of sputum samples.

### Molecular characteristics of MRSA ST630 isolates

**Fig 1:**
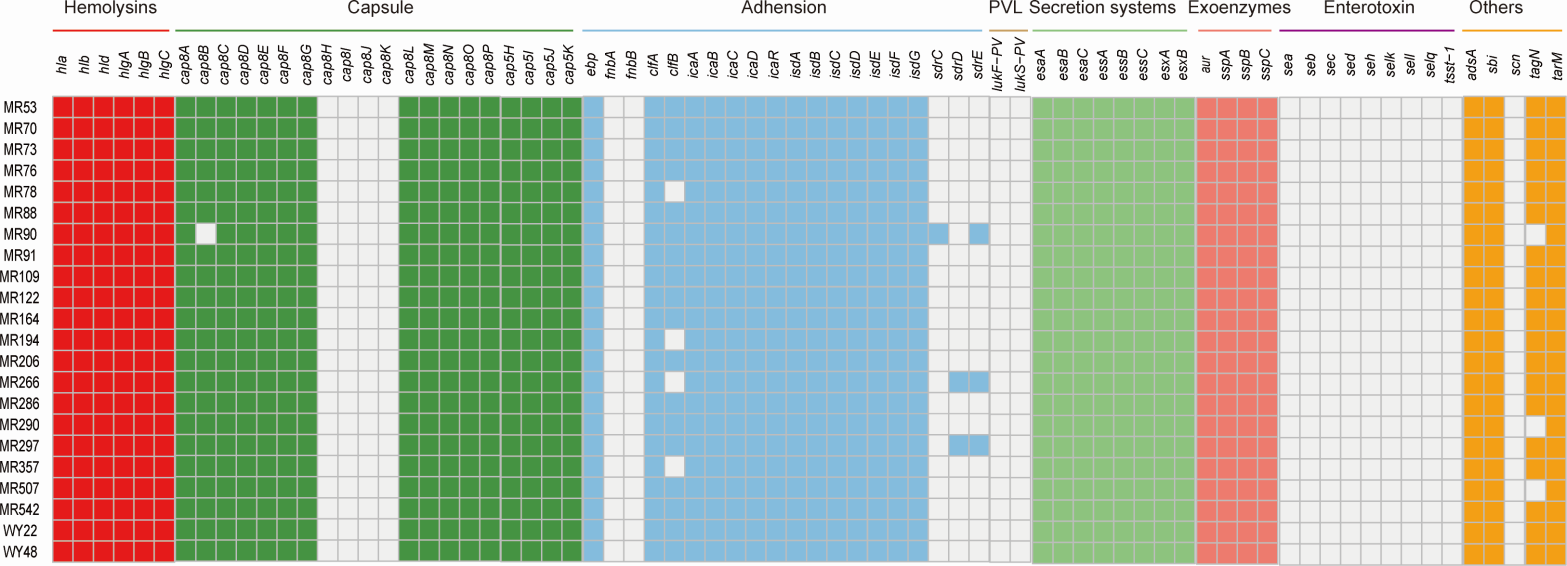
Virulence gene profiles of the 22 ST630 *S. aureus* isolates. Colored blocks represent the presence of genes and white blocks represent the absence. The horizontal color bar (from left to right) represents genes associated with hemolysins, capsule, adhesion, PVL, secretion systems, exoenzymes, enterotoxins, and other virulence genes.

SCCmec type V was detected in 50% of the isolates (11/22), while one ST630 isolate carried SCCmec IV, representing 4.5% (1/22), and the remaining 10 ST630 isolates were undetermined. The most prevalent spa type among all ST630-V isolates was t4549, representing 54.5% (6/11), while two ST630-V isolates belonged to spa types t377 and t2196, respectively, and the remaining three isolates were uncertain. As shown in Fig. 1, all ST630 isolates carried the *hla*, *hlb*, *hlg*, and *hld* genes associated with hemolysin and cap5H, cap5I, cap5J, cap5K associated with Capsule polysaccharide synthesis. Besides that, secretion system including *esaA*, *esaB*, *esaC*, *essA*, *essB*, *essC*, *esxA*, and *esxB* genes and exoenzyme consisting of *aur*, *sspA*, *sspB*, and *sspC* genes were also carried by all ST630 isolates. By contrast, all ST630 isolates lacked the *lukS/F-PV* genes encoding Panton-Valentine leukocidin (PVL) along with *sea*, *seb*, *sec*, *sed*, *seh*, *selk*, *sell*, *selq*, and *tsst-1* genes relevant to enterotoxin. All 22 ST630 strains in this study carried tarM, while only 19 ST630 strains carried tagN related to teichoic acid synthesis.

### Antimicrobial resistance profiles of MRSA ST630 isolates

**Fig 2:**
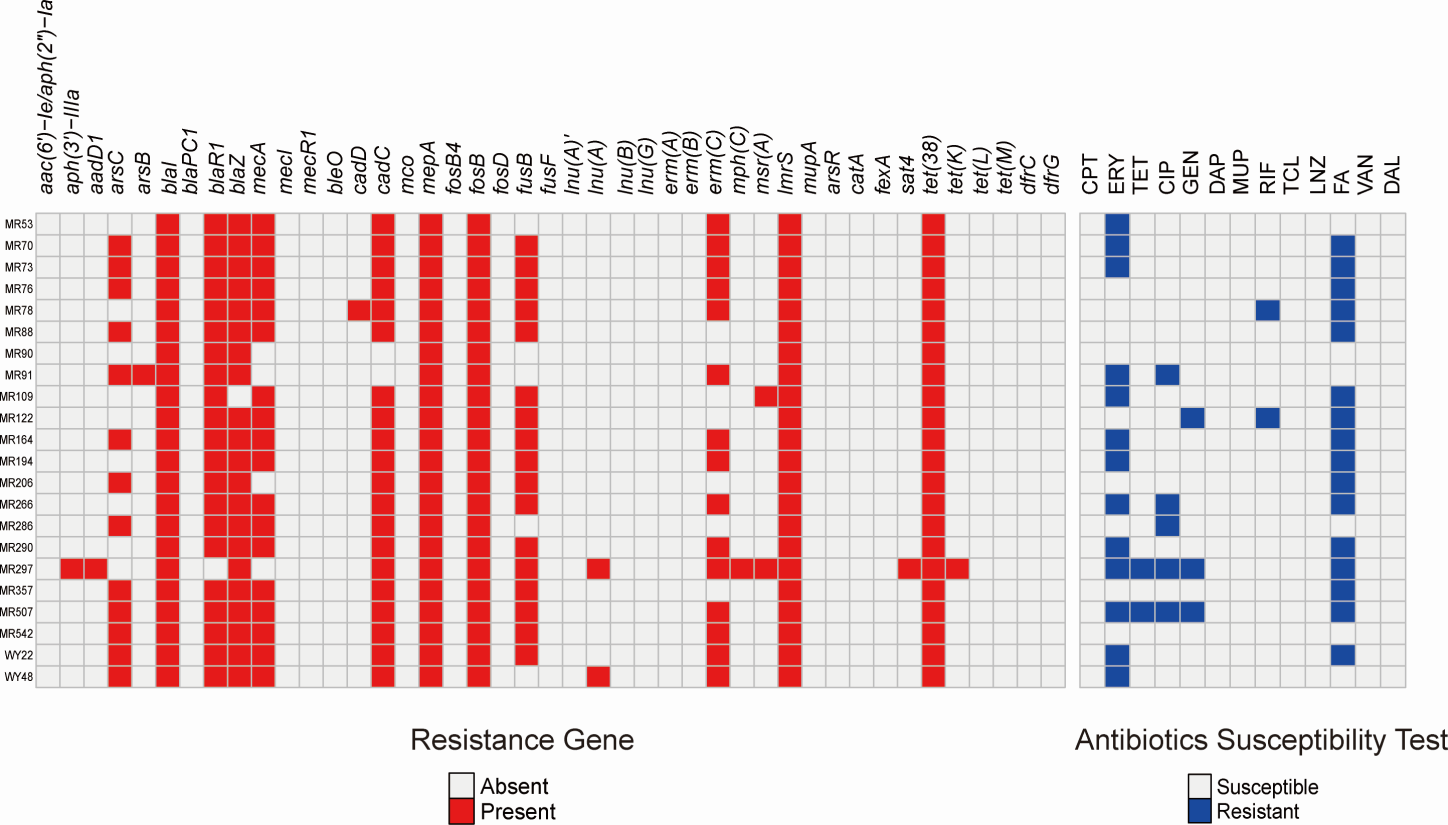
Antibiotic resistance genes (left) and antibiotic susceptibility profiles (right) of 22 ST630 *S. aureus* isolates that were collected in this study.

The antimicrobial susceptibility results are demonstrated in Fig. 2 (right). All ST630 isolates were susceptible to ceftaroline, daptomycin, mupirocin, teicoplanin, linezolid, vancomycin, and dalbavancin. The resistance rate of fusidic acid was the highest (16/22, 72.7%). As listed in Fig. 2 (left), all isolates harbored an antimicrobial resistance gene conferring the ST630 isolates resistance phenotype to β-lactam (*blal*), fosfomycin (*fosB*), tetracycline (*tet38*), and multidrug efflux transporter (*mepA* and *lmrS*). The *blaI* gene, which confers β-lactam resistance, was supported by phenotypic resistance results for our ST630-MRSA isolates.

### Phylogenetic and comparative genomic analysis

**Fig 3:**
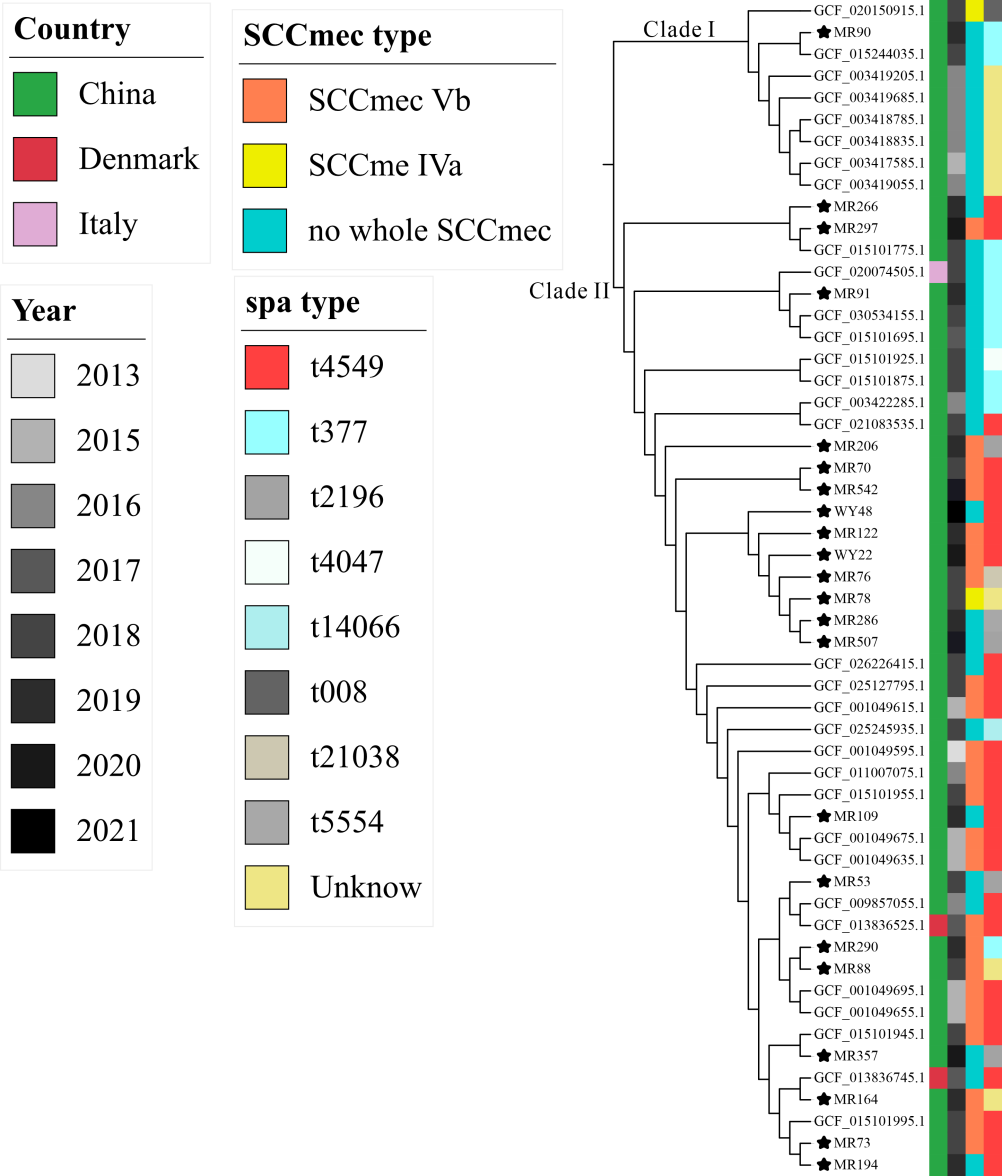
Phylogenetic analysis of ST630 *S. aureus* isolates.

To explore the evolutionary history of ST630 isolates, a phylogenetic tree was constructed based on core SNPs. This included our collection of 22 ST630 isolates and an additional 31 ST630 isolates from NCBI RefSeq, sampled up to October 2023. As illustrated in Fig. 1, the earliest identified ST630 isolates were traced back to China in 2013, with a significant majority of the isolates (50 out of 53, 94.34%) being traced to this region. The remainder, three ST630 isolates, were obtained from Denmark (*n* = 2) and Italy (*n* = 1), indicating the international emergence of ST630 isolates. The 53 ST630-MRSA isolates bifurcated into two distinct clades; notably, Clade I consisted of eight isolates, all of which lacked a complete SCCmec element (Fig. 3). These eight ST630-MRSA isolates in Clade I exhibited an average divergence of 193 core SNPs, ranging from 1 to 306 SNPs, indicative of the diversification among MRSA ST630 clonotypes. Within the more populous Clade II, approximately half of the strains (23 out of 45, 51.11%) were identified as SCCmec type Vb, with a solitary strain characterized as type SCCmec IVa. Interestingly, within the 22 ST630-MRSA isolates examined, nearly half (45.5%) did not harbor a complete SCCmec element. The 45 ST630-MRSA isolates in Clade II displayed an average of 294 core SNPs, with a distribution ranging from 0 to 1,109. Thus, the result suggests that ST630-MRSA likely originated in China and has since become widespread within the region.

### Hemolysin activity determination

**Fig 4:**
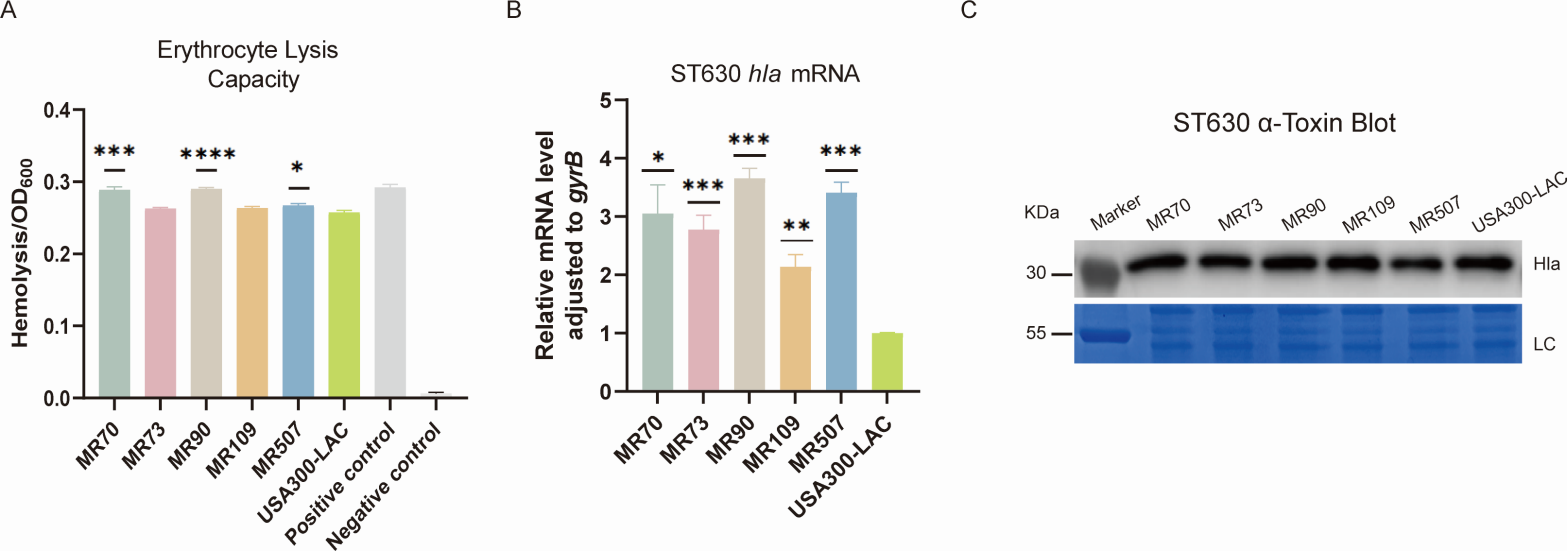
Comparison of the hemolysis ability and the mRNA and protein level of *hla* in the ST630 *S. aureus* isolates. (**A**) Erythrocyte lysis capacity of the randomly selected five ST630 isolates and the USA300-LAC strains. Triton X-100 served as the positive control, and 0.9% (wt/vol) NaCl was set as the negative control. The absorbance of each sample was measured at 600 nm. (**B**) The transcriptional level of *hla* in the randomly selected five ST630 isolates. Transcript levels were normalized to *gyrB* transcript level. (**C**) Western blot assay was used to assess the secretion of α-toxin in the randomly selected five ST630 isolates. The total bacterial proteins served as loading control (LC). Molecular weights of the protein marker were indicated on the left. *P < 0.05; **P < 0.01; ***P < 0.001; and ****P < 0.0001.

To analyze the virulence characteristic of ST630 collected in this study, we randomly selected five strains for subsequent phenotype experiments according to the results of the phylogenetic analysis. *S. aureus* secretes numerous cytolytic toxins, which target and kill mammalian cells by disrupting their plasma membranes. The cytolytic activity of MRSA contributes to the pathogenesis of MRSA by assisting it evade phagocytic cell-mediated killing. As shown in Fig. 4A, MR90 had the highest hemolysis ability among these MRSA strains (*P* < 0.0001), along with MR70 (*P* < 0.001) and MR507 (*P* < 0.05), which displayed higher levels of hemolysis capacity against erythrocytes in comparison with the USA300-LAC strain. The hemolytic abilities of the MR73 and the MR109 strain were comparable. It should be noted that the hemolytic activity of most (72.73%, 16/22) ST630-MRSA strains in this study were higher than that of USA300-LAC or equivalent (Fig. S1).

### RT-qPCR and western blot

We next tested whether ST630 isolates exhibited high hemolysis activity due to the high *hla* expression by RT-qPCR and western blot. A higher expression was observed obviously in all five ST630 strains than the USA300-LAC strain (Fig. 4B). The *hla* mRNA expression of MR90, MR507, and MR70 were considerably increased by 3.65-fold (*P* < 0.001), 3.41-fold (*P* < 0.001), and 3.05-fold (*P* < 0.05), respectively. Apart from this, the levels of *hla* mRNA in MR73 and MR109 were increased by 2.78-fold (*P* < 0.001) and 2.14-fold (*P* < 0.01) separately. The western blot assay results of ST630 isolates demonstrated that except for MR507, which produced a slightly weaker level of α-toxin in comparison to the USA300-LAC strain, the other four stains were equivalent to the USA-LAC strains (Fig. 4C). The results manifested that these strains possessed excellent virulence potential and corresponded to the above-described phenotype.

### H_2_O_2_ killing and whole-blood killing assay

**Fig 5:**
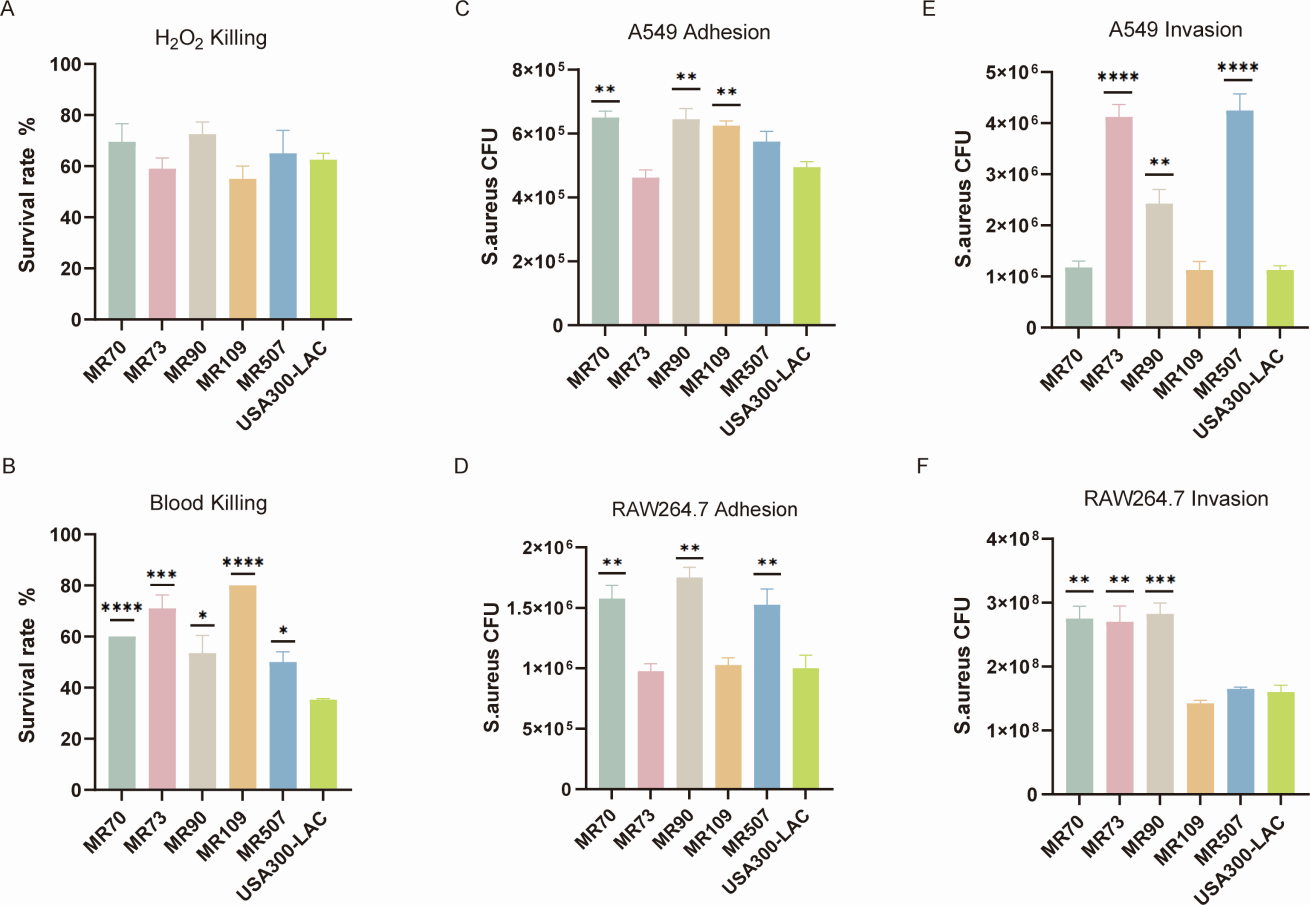
Comparison of the virulence of the ST630 *S. aureus* isolates *in vitro*. (**A**) The susceptibility of randomly selected five ST630 isolates to killing by H_2_O_2_. (**B**) The susceptibility of five ST630 isolates to killing by human whole blood. (**C**) Cell adhesion assays in A549 cells of the randomly selected five ST630 isolates. (**D**) Cell adhesion assays in RAW264.7 cells of the five ST630 isolates. (**E**) Cell invasion assays in A549 cells of the five ST630 isolates. (**F**) Cell invasion assays in RAW264.7 cells of the five ST630 isolates. *P < 0.05; **P < 0.01; ***P < 0.001; and ****P < 0.0001.

To test the ability of the ST630 strain to resist oxidative killing and whole-blood killing, we performed the H_2_0_2_ killing and whole-blood killing assay. In terms of the ability to resist H_2_0_2_ oxidative killing, all five ST630 isolates were comparable to the USA300-LAC strains (Fig. 5A). Conspicuously, MR109 exhibited the highest resistance to the whole blood, its survival rate reached as high as 80% (*P* < 0.0001). Besides that, MR73 displayed an excellent survival rate with a resistance to the whole blood of 71% (*P* < 0.001). Furthermore, the survival rates of MR70, MR90, and MR507 which were 60% (*P* < 0.0001), 53.5% (*P* < 0.05), and 50% (*P* < 0.05), respectively, were also higher than the USA300-LAC strain which was 35.25% (Fig. 5B).

### Cell adhesion and invasion experiment

MRSA infections have continued to be a challenge in hospital environments. Bacterial adhesion is the first crucial step in infections, as it facilitates colonization and subsequent invasion across the mucosal barrier. This led us to examine the adhesion capabilities of ST630 isolates. To further explore the adhesion capabilities of ST630 isolates, we performed the cell adhesion assay using A549 and RAW264.7 *in vitro*. As shown in Fig. 5C, the A549 adhesion abilities of MR70, MR90, and MR109 isolates were stronger than the USA300-LAC strains (6.50 × 10^5^ CFU, 6.45 × 10^5^ CFU, and 6.25 × 10^5^ CFU separately vs 4.95 × 10^5^ CFU, *P* < 0.01). Likewise, Fig. 5D demonstrated that MR70, MR90, and MR507 isolates showed greater adhesion ability against RAW264.7 cells than the USA300-LAC strains (1.58 × 10^6^ CFU, 1.75 × 10^6^ CFU, and 1.53 × 10^6^ CFU separately vs 1.00 × 10^6^ CFU, *P* < 0.01). *S. aureus hla* is closely associated with the pathogenic mechanisms of host colonization and invasive disease. Therefore, we further evaluated the invasive effect of ST630 isolates on A549 and RAW264.7 cells (Fig. 5E and F). As expected, both MR73 and MR507 displayed an extremely higher invasive capacity against A549 cells than the USA300-LAC strains (4.13 × 10^6^ CFU and 4.25 × 10^6^ CFU separately vs 1.12 × 10^6^ CFU, *P* < 0.0001). Except for these, the A549 cell invasion ability of the MR90 strain was also stronger than the USA300-LAC strains (2.43 × 10^6^ CFU vs 1.12 × 10^6^ CFU, *P* < 0.01). Similarly, the RAW264.7 cell invasion ability of the MR90 was higher than the USA300-LAC strains (2.83 × 10^8^ CFU vs 1.50 × 10^8^ CFU, *P* < 0.001). Moreover, MR70 and MR73 demonstrated a more wonderful invasion level against RAW264.7 cells compared to the USA300-LAC strains (2.75 × 10^8^ CFU and 2.70 × 10^8^ CFU separately vs 1.50 × 10^8^ CFU, *P* < 0.01).

### Mouse skin abscess model

**Fig 6:**
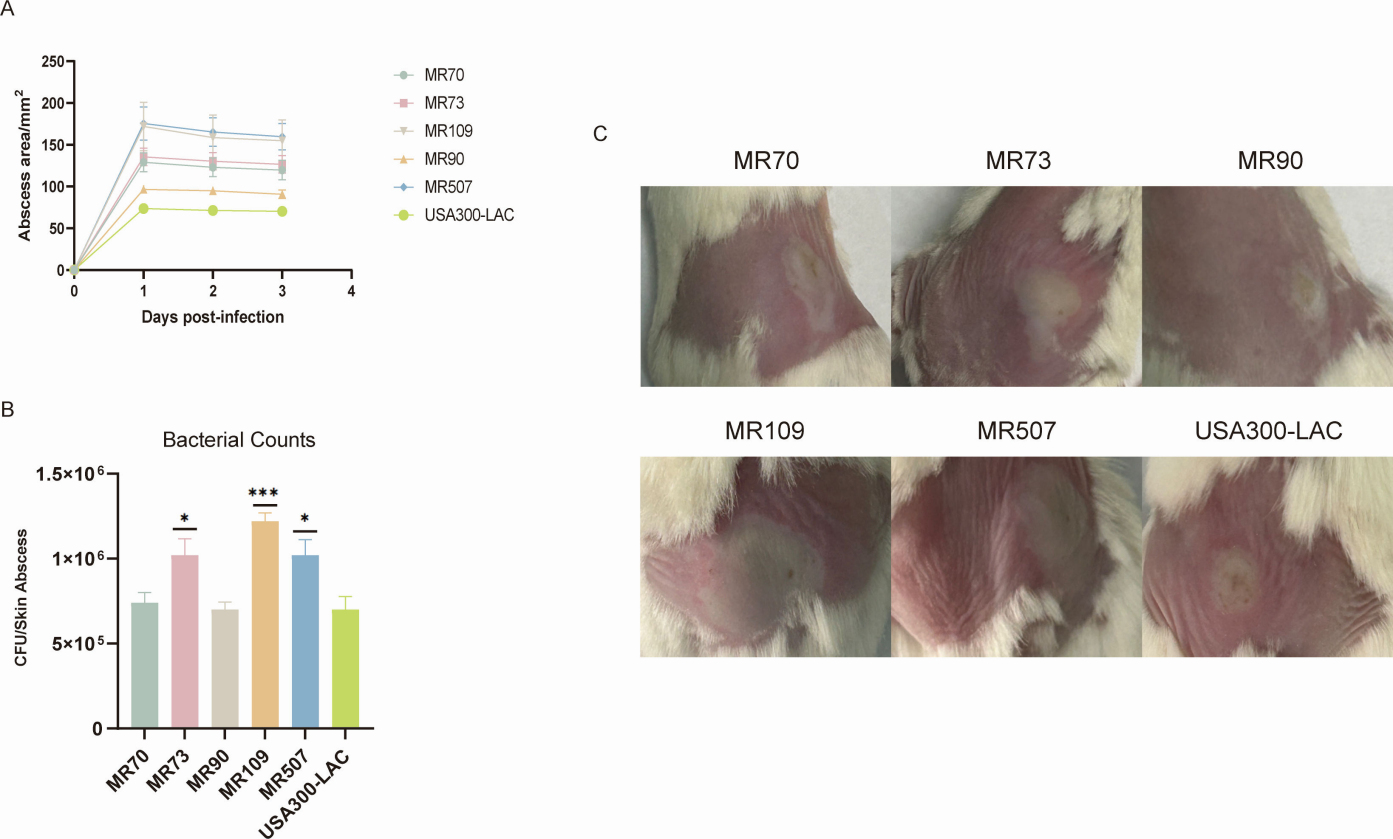
Comparison of the virulence of the ST630 *S. aureus* isolates *in vivo*. (**A**) Daily changes of skin abscess area in mice before and after treatment. Randomly selected five ST630 isolates were used for infections in mouse back skin. USA300-LAC was selected and compared with ST630. (**B**) Comparison of bacterial colonies in mice skin. Monitored the lesion and abscess sizes daily with a caliper using the formula *A* = π × (*L*  ×  *W*)). (**C**) The image of representative abscesses. *P < 0.05 and ***P < 0.001.

After observing the ST630 isolates on the activity of hemolysis, and the expression levels of *hla* and α-toxin, we sought to examine its virulence *in vivo* using a mouse skin abscess model. After subcutaneously inoculated with 100 µL PBS containing 1 × 10^7^ bacterial cells for 48 h, the abscess area was shown in Fig. 6C. The abscess size arising from all five ST630 isolates were significantly larger than the USA300-LAC strains (Fig. 6A). On the first day post-infection, it is noteworthy that the abscess size induced by MR507 was the largest, up to 176 mm^2^, followed by the abscess size caused by MR109 which was 172 mm^2^. As shown in Fig. 6B, the mice infected with MR73 (*P* < 0.05), MR109 (*P* < 0.001), and MR507 (*P* < 0.05) isolates had significantly higher CFU counts compared to the USA300-LAC strains. Additionally, the bacterial counts of mice infected with MR70 and MR90 were comparable to the USA300-LAC. Taken together, our data indicated that the Chinese MRSA ST630 isolates were highly virulent.

## DISCUSSION

*S. aureus,* especially MRSA, has been recognized as a significant threat to public health in the past few decades (21–23). MRSA is notorious for its ability to acquire resistance to multiple antibiotics (24–26) and its high virulence (27–29). In recent years, the epidemiological landscape of MRSA has been changing, with the emergence of new clones, such as ST630, which has become a prevalent strain in both Denmark and China (10). In a tertiary hospital in China, the proportion of ST630-MRSA among ST630 *S. aureus* clones dramatically increased from 0% in 2008 to 57% in 2017 (8). Despite this alarming trend, the genomic characteristics and evolutionary history of ST630-MRSA in China remain largely uncharacterized. In this study, we conducted a comprehensive genomic and phenotypic analysis of 22 ST630 isolates collected from six provinces in China. These isolates were primarily obtained from pus and wound secretions, with a majority originating from Jiangxi province. Notably, all isolates harbored diverse virulence and antibiotic resistance genes, with 50% of them carrying the SCCmec V. This finding is consistent with previous reports on the prevalence of SCCmec V among ST630 strains (9, 11).

Mikkelsen et al. (9) reported the prevalence of CRISPR-Cas systems in clinical MRSA strains in Denmark and showed that type III-A CRISPR-Cas systems were present in more than half of the examined strains belonging to the emerging clone ST630. Interestingly, the CRISPR-Cas locus in the ST630 strain was located within the SCCmec cassette that, in MRSA strains, carried the mecA gene encoding the alternative penicillin-binding protein providing methicillin resistance. The CRISPR-Cas system of MRSA strains such as ST630 could enhance their defense against phage attacks, which was an important advantage for the survival of bacteria in natural environments or under antibiotic pressure. It might also indirectly affect the prevalence and virulence of strains by influencing the horizontal transmission of genes. Moreover, the extensive homology of cas genes and CRISPR spacers between *S. aureus* ST630 and CoNS suggests a possible recent exchange of CRISPR-cas and SCCmec between these two species (11). This finding, along with the acquisition of GroP-WTA biosynthesis genes from CoNS by ST630 strains, indicates a potential evolutionary connection between ST630 and CoNS, which may contribute to the adaptation and success of this clone.

Generally, bacterial virulence is a multi-factorial process that requires the use of a variety of components, many of which are coordinately regulated to allow the organism to adapt to the host environment and become successful pathogens. Genetic annotation showed that all the ST630 isolates were hemolysin-positive; it was consistent that randomly selected ST630 isolates had high hemolysis ability. Staphylococcal α-toxin is one of the key virulence determinants in the pathogenesis of *S. aureus* (30–33), encoded by the *hla* gene and is associated with skin and soft tissue infections (SSTIs) and sepsis (33–36). As expected, not only the mRNA expression of *hla* but also the protein level of α-toxin in ST630 isolates was higher than the USA300-LAC strains.

Data in mounting numbers suggested that *S. aureus* could adhere and invade different types of host cells, which contributed to evading the host immune defense (37). Bacterial adhesion and invasion assay was carried out to assess the binding efficiency of *S. aureus* to A549 and RAW264.7 cells separately. In this study, higher cell adhesion capabilities were found for ST630 isolates. These isolates could persist and were difficult to eradicate due to their strong adherence to the human lung epithelial cells and murine macrophage cells. The invasion of host cells by *S. aureus* ultimately led to the formation of cytoplasmic reservoirs, where bacteria were protected from the immune cell and antibody-mediated immune response, as well as the effects of most antimicrobial agents (38–40). This bacterial sanctuarization made successful treatment even more challenging and paved the way for infection relapse (41–43). Additionally, the high invasion capacity of ST630 isolates might allow it to survive within the host for a long time, contributing to the dissemination of this clone.

The skin barrier protects the human body from invasion by exogenous and pathogenic microorganisms. The Gram-positive bacterium *S. aureus* was a frequent colonizer of the skin and mucosae of the human host (44, 45) and was often the cause of SSTIs (46–48); as shown in our mouse skin abscess model, the abscess size arising from all the five ST630 isolates were significantly larger than the USA300-LAC strains. Likewise, the increase in bacterial counts of skin abscesses pointed out the high virulence of ST630 isolates. MRSA USA300 was the predominant cause of SSTIs (49, 50) with α-toxin contributing to superficial and invasive disease, together with interfering with host immunity during skin infections and recurring disease (31, 51). The findings of this study have significant implications for the management and control of MRSA infections in China. The high prevalence of SCCmec V and the hypervirulent nature of ST630 strains highlight the urgent need for enhanced surveillance and infection control measures to prevent the further dissemination of this emerging clone. Additionally, the potential role of ST630 as a hub for the exchange of mobile genetic elements between *S. aureus* and CoNS warrants further investigation, as it may contribute to the emergence of novel pathogenic strains with increased antibiotic resistance and virulence.

In conclusion, our study provides a comprehensive characterization of the genomic and phenotypic features of the emerging ST630-MRSA clone in China. The acquisition of diverse antibiotic resistance and virulence genes, coupled with the hypervirulent traits observed in phenotypic assays, underscores the potential threat posed by this clone to public health. Further research is needed to elucidate the molecular mechanisms underlying the success and adaptability of ST630-MRSA and to develop effective strategies for the prevention, diagnosis, and treatment of infections caused by this emerging clone. Our findings contribute to a better understanding of the evolving epidemiology of MRSA in China and highlight the importance of continued surveillance and research to combat the ever-growing threat of antibiotic resistance and virulence in *S. aureus*.

## Data Availability

The Illumina sequences of the 22 ST630 isolates are available in NCBI (accession number: PRJNA1125655).

## SUPPLEMENTAL MATERIAL

Figure S1 msystems.00664-24-s0001.rtf The hemolytic activity of all 22 strains in this study.

ASM does not own the copyrights to Supplemental Material that may be linked to, or accessed through, an article. The authors have granted ASM a non-exclusive, world-wide license to publish the Supplemental Material files. Please contact the corresponding author directly for reuse.
