# Supplementary material for: Phenotypic and genomic analysis of the hypervirulent methicillin-resistant Staphylococcus aureus ST630 clone in China
Source: mSystems. 2024 Aug 19;9(9):e00664-24. doi: 10.1128/msystems.00664-24 (PMC11406941; doi:10.1128/msystems.00664-24)
Supplement: Figure S1 — The hemolytic activity of all 22 strains in this study. [file msystems.00664-24-s0001.rtf]

Supplementary Figure S1
The hemolysin activity determination assay for all the 22 strains and the USA300-LAC strains. TritonX-100 served as the positive control, and 0.9% (w/v) NaCl was set as the negative control. Measured the absorbance of each sample at 600 nm. *P<0.05; **P<0.01; ***P<0.001 and ****P<0.0001.
